# Analysis of pan-African Centres of excellence in health innovation highlights opportunities and challenges for local innovation and financing in the continent

**DOI:** 10.1186/1472-698X-12-11

**Published:** 2012-07-27

**Authors:** Solomon Nwaka, Alexander Ochem, Dominique Besson, Bernadette Ramirez, Foluke Fakorede, Sanaa Botros, Uford Inyang, Charles Mgone, Ivan Adae-Mensah, Victor Konde, Barthelemy Nyasse, Blessed Okole, Anastasia Guantai, Glaudina Loots, Peter Atadja, Peter Ndumbe, Issa Sanou, Ole Olesen, Robert Ridley, Tshinko Ilunga

**Affiliations:** 1 African Network for Drugs and Diagnostics Innovation, United Nations Economic Commission for Africa, Addis Ababa, Ethiopia; 2 UNICEF/UNDP/World Bank/WHO Special Programme for Research and Training in Tropical Diseases (TDR), World Health Organization, Geneva, Switzerland; 3 Theodore Bilharz Research Institute, Cairo, Egypt; 4 Fordsin Pharmacare Systems Ltd, Abuja, Nigeria; 5 European and Developing Countries Clinical Trials Partnership, The Hague, Netherlands; 6 Department of Chemistry, University of Ghana, Legon, Ghana; 7 United Nations Economic Commission for Africa, Addis Ababa, Ethiopia; 8 University of Yaoundé, Yaoundé, Cameroon; 9 Technology Innovation Agency, Department of Science and Technology, Cape Town, South Africa; 10 University of Nairobi, Nairobi, Kenya; 11 Department of Science and Technology, Cape Town, South Africa; 12 Novartis Institutes for BioMedical Research, Shanghai, China; 13 Regional Office for Africa, World Health Organization, Brazzaville, Congo; 14 European Commission, DG Research, Brussels, Belgium; 15 University of Malawi, Box 278, Zomba, Malawi; 16 Health Development Consultant, Abidjan, Ivory Coast

## Abstract

A pool of 38 pan-African Centres of Excellence (CoEs) in health innovation has been selected and recognized by the African Network for Drugs and Diagnostics Innovation (ANDI), through a competitive criteria based process. The process identified a number of opportunities and challenges for health R&D and innovation in the continent: i) it provides a direct evidence for the existence of innovation capability that can be leveraged to fill specific gaps in the continent; ii) it revealed a research and financing pattern that is largely fragmented and uncoordinated, and iii) it highlights the most frequent funders of health research in the continent. The CoEs are envisioned as an innovative network of public and private institutions with a critical mass of expertise and resources to support projects and a variety of activities for capacity building and scientific exchange, including hosting fellows, trainees, scientists on sabbaticals and exchange with other African and non-African institutions.

## Introduction

The African continent bears the greatest burden of disease in the world today [1,2], but it has no mechanism to ensure sustainable access to the health tools needed by its people. Investment in health research and innovation is a major factor in overcoming the high disease burden in the developing world especially Africa [3-5]. A number of international and pan-African reports and actions such as the Commission on Health Research for Development [6], the Accra Plan of Action [7], the Abuja declaration of 2001 by African leaders [8], and a number of African Ministerial Declarations [9,10], have stressed the need to invest in health and R&D capacity for diseases that are predominant in developing countries especially Africa [3,11]. The global strategy and plan of action on public health innovation and intellectual property (GSPOA) approved through World Health Assembly resolutions also underlines the need to invest in R&D innovation and capacity building in developing countries [4,12]. Although meeting the necessary health and health R&D investment targets in Africa remains a challenge for most African countries and development partners, there are promising signs of improvement. Some African countries such as South Africa, Kenya and Uganda are committing about 1% of their gross domestic product (GDP) to R&D activities [13], while development partners are increasingly discussing ways to enhance support for research and capacity building in Africa [4,7].

Despite these developments, the current funding streams for health research in Africa are still fragmented and characterized by a number of small and short term grants that are not always contributing to long-term development of the health research system [14,15] and it has particularly been argued that donor driven science can lead to biased research agendas towards donor interests in certain countries, activities or specific diseases [16-18].

In addition, there is a lack of reliable data on capacity for health research as well as limited knowledge about where the real bottlenecks are, further underlining the need for more institutional, systems and capacity evaluation [15]. These observations sometimes paint a pessimistic view about the prospects of implementing a robust health products R&D in Africa. Indeed, new data are now emerging on the available capacity in the continent and how this capacity can be leveraged to contribute in solving Africa’s health challenges. The mapping of the health R&D landscape in Africa, implemented in the course of the development of the African Network for Drug and Diagnostics Innovation (ANDI, http://www.andi-africa.org), suggests that capacity for product R&D and innovation exists in the continent [3,19-21]. However, this capacity is not effectively utilized to fill gaps and solve Africa’s health problems due to the lack of collaboration within Africa, lack of sustainable funding and coordination of existing research efforts as well as governance and other issues. Other reports have also reached similar conclusions [22-24].

Another recent report has stressed that the current global funding arrangements for public health need to go beyond provision of treatments, but should also focus on building requisite health research and development infrastructure in the African continent [13]. The report argues that this will position African institutions to take advantage of not only globally available health technologies and products, but also invest in research institutions that are both knowledge-based and oriented towards product development. Despite these technical, policy and political reports, little has materialized in terms of structured implementation of concrete health innovation activities in Africa, and existing capacity is not always leveraged to support health research and development to generate health products and evidence for policy.

As part of the implementation of ANDI activities and consistent with its vision to leverage available capacity in the continent, we initiated the identification and recognition of African institutions with capacity in the various areas of the health product innovation value chain, including basic research, discovery, development, manufacturing and commercialization. We sought to evaluate the capacity, competency and funding of African institutions through a transparent workflow guided by a set of criteria and review process. The goal was to identify a pool of pan-African Centers of Excellence (CoEs) in health innovation with necessary capacity and infrastructure to support the implementation of projects and capacity building in the continent. We describe the process that led to the identification of 38 Centres of Excellence in health innovation, and how these centres will contribute to achieving the ANDI vision of creating a sustainable platform for health innovation in Africa and providing solutions to the continents health challenges.

## Methods

### Call for Centres of Excellence (CoEs) and response

**Figure 1:**
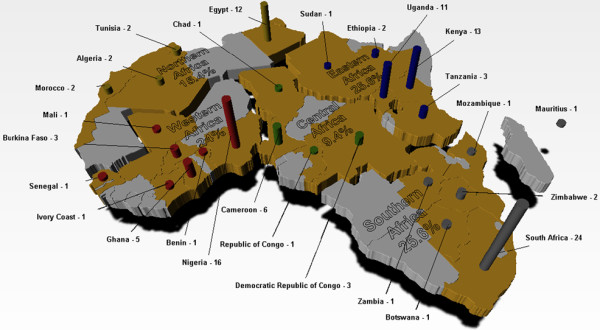
**Continental mapping of applications received from the call for Centers of Excellence.** This figure represents the Continental, sub-regional and national coverage of applications submitted in response to the call for Centres of Excellence by ANDI. Represented in gold colour are all countries from which at least one application was submitted and sub-regional grouping of countries was done accordingly to the African Development Bank definition (http://www.afdb.org/en/countries/). One or two countries per region are responsible for the bulk of applications in each region, for example, Egypt in Northern Africa, Kenya and Uganda in Eastern Africa, Republic of South Africa from Southern Africa, Nigeria in Western Africa and Cameroon in Central Africa.

The identification of potential centers of excellence was implemented through an open call for applications [25]. Considering the different languages in Africa, the call was prepared in both English and French, and broadly publicized at relevant meetings in Africa and overseas, various websites as well as through mailing lists, e-mails and advertisement in a major scientific journal. At the deadline of the call, 117 applications from institutions across Africa were received (Figure 1).

Some of the 117 applications requested to be considered as centers of excellence in multiple competency areas, thereby making the number of applications based on competencies to be 132. The country distribution, regional spread and the various competencies across the product R&D value chain for the applications received are shown on Table 1 and Figure 1. The geographic spread of CoE applications across the different African countries and regions, demonstrates the pan African significance of this process. Applications were received from 26 countries covering the five regions of Africa. Similar numbers of applications were received from Southern, Eastern and Western Africa as follows, 25.6%, 25.6% and 24.0% respectively; while Northern and Central Africa had lesser numbers with 15.4% and 9.4% of the applications respectively (Figure 1). The fact that about 50% of African countries were represented in this first call for CoE is indicative of the level of interest in the process within the continent. We believe that with broader advertisement of future calls for application, many more countries will be covered.

**Table 1:** ANDI call for Center of Excellence results

**Countries**	**Applications**	**Regions**	**Functional competency R&D area**							
			**Basic Research**	**Product Discovery**	**Traditional Medicines**	**Preclinical development**	**Clinical development**	**Manufacturing**	**Product Evaluation**^**1**^	**Other**^**2**^
Algeria	**2** (3)	North	-	-	-	-	-	-	1	2
Egypt	**12** (13)		2	5	1	-	-	1	2	2
Morocco	**2** (4)		-	2	-	-	-	-	-	2
Tunisia	**2**		-	1	-	-	-	-	1	-
Ethiopia	**2**	East	-	-	1	-	-	-	1	-
Kenya	**13**		2	1	2	2	2	1	1	2
Sudan	**1**		-	1	-	-	-	-	-	-
Tanzania	**3**		1	1	-	-	-	1	-	-
Uganda	**11** (12)		1	1	1	-	4	1	2	2
Botswana	**1**	South	-	-	-	-	-	1	-	-
Mauritius	**1**		-	1	-	-	-	-	-	-
Mozambique	**1**		-	-	-	-	1	-	-	-
South Africa	**24**		8	9	-	1	1	1	-	4
Zambia	**1**		-	-	-	-	-	-	1	-
Zimbabwe	**2** (4)		1	2	-	-	1	-	-	-
Benin	**1**	West	-	-	-	-	-	-	-	1
Burkina Faso	**3**		-	2	-	-	1	-	-	-
Côte d'Ivoire	**1**		-	-	-	-	-	-	-	1
Ghana	**5** (6)		1	2	1	-	1	1	-	-
Mali	**1**		-	-	-	-	1	-	-	-
Nigeria	**16** (21)		-	7	8	2	2	-	-	2
Senegal	**1**		-	-	1	-	-	-	-	-
Cameroon	**6** (7)	Central	1	3	2	-	-	-	-	1
Chad	**1** (2)		-	-	-	-	-	-	1	1
Democratic Republic of Congo	**3**		-	1	-	-	-	-	1	1
Republic of Congo	**1**		-	-	-	-	-	-	-	1
Total	**117** (132)		18	39	17	5	14	7	11	22

^1^ Activities related to evaluation of new or already registered products or available technology including implementation research, or diagnostics performance evaluation etc.

^2^ Epidemiology, Health Systems, Ethics, IP management, Knowledge & data management.

This table shows specific number of applications received by countries and regions as well as functional R&D competency areas that were covered by application received. Total number of applications per competency area received is 132, which is higher than the total number of individual applications which is 117. The reason for this is that some applications did apply to be considered for two or more competency areas within their single application. Numbers in the *Applications* column represent the number of individual application per country (in bold, 117 in total) and the number of competency areas covered by the applications (in brackets, 132 in total). Please note that the competency areas outlined (mirror the call), and these were all covered in the applications received and cover the different part of the product R&D value chain.

### Review and assessment of applications for CoEs

The 117 applications were evaluated using a 4-step review process based on defined qualitative and quantitative criteria [25]. These criteria include: availability of infrastructure and equipment for the specific research area; number of staff working in the area; track record of the technical staff; productivity of the institution over a period of time as measured by publications in peer reviewed journals or patents or products discovered or developed; availability and access to good communication tools including internet, website, telephone etc., as well as financial sustainability of the institution as evidenced by budget allocation over a 3-year period (2008–2010).

The review of the applications for CoEs was implemented in different phases as follows:

1) Phase 1 review involved assessment by peers: this involved heads of the applying institutions in a manner that avoided conflict of interest or having peers review their own applications or applications from collaborating institutions. This first review helped to isolate the 76 applications identified as scoring at least 70 out of 100 points which would enter the second reviewing phase.

2) Phase 2 review involved evaluation by the ANDI Scientific and Technical Advisory Committee (STAC), which is an important part of ANDI’s structure [3]. Shortlisted applications were sent electronically to STAC members for review and scoring without revealing the scores from phase 1. Following the feedback from this review all scores from STAC were tallied alongside the scores from the phase 1 review and the overall score sheet was sent back to STAC electronically in preparation for phase 3.

3) 1Phase 3 review involved a face to face meeting of STAC to discuss the 70 applications that scored over 70 points out of a total of 100 points after second review, with the objective of recommending those that met most of the criteria, scoring at least 80 points, as potential CoE. This resulted in the identification of 33 centers, which entered phase 4.

4) Phase 4 review involved verifications of the information presented by short listed 33 centers. Following extensive electronic assessment of these centers including referee reports and interviews, a total of 32 centers were recommended by STAC as ANDI CoE in health innovation in Africa. At this stage, STAC agreed to further assess applications from manufacturers based on additional criteria established to determine their manufacturing status, including any national or international certification by relevant authorities that the centers may have attained. Evaluation of this information resulted in accepting an additional six CoEs from the manufacturers sector making the total number of CoEs 38 (Table 2). The STAC further recommended that the designation of CoE be granted by ANDI for a period of 5 years with a mid-term review that will involve site visits and evaluation of CoEs using an outcome metrics. This outcome metrics is being developed in consultation with the CoEs. Furthermore, new calls for CoEs will be launched intermittently by ANDI to identify new Centres that meet the CoE criteria or fill a specific gap that has been identified by ANDI.

**Table 2:** Selected ANDI Centers of Excellence in Health Innovation

**Applying Institution and country**	**Applying department or unit**	**Name of CoE**	**Institution contact and website**	**CoE Regional location**
Institute of Medical Research and Medicinal Plants Studies	Departments Of Phytochemistry, Toxicology/Pharmacology, Botany/ Traditional Medicine and Pharmaceutical Technology	ANDI Centre of Excellence in Traditional Medicine Research	**Dr Essame Oyono**	Central
*Cameroon*			Dr Agbor	
			www.minresi.net	
University of Buea	Faculty Of Science (Departments Of Chemistry + Biotechnology Unit)	ANDI Centre of Excellence for Onchocerciasis Drug Research	**Prof Titanji**	Central
			Dr Cho-Ngwa	
*Cameroon*			Prof Efange	
			http://www.ubuea.net	
National Center for Research	Medicinal And Aromatic Plants Research Institute, Tropical Medicine Research Institute	ANDI Centre of Excellence for Drug Discovery & Diagnostic Innovation	**Prof Ahmed**	Eastern
*Sudan*				
			http://www.ncr.sd	
Institute of Primate Research	Institute Of Primate Research - (Tropical & Infectious Diseases, Drugs Program/ Natural Products Platform, Reproductive Health & Biology, Non-Communicable Diseases, Animal Sciences)	ANDI Centre of Excellence in Pre-clinical Research	**Dr Kariuki**	Eastern
*Kenya*				
			http://www.primateresearch.org	
Kenya Medical Research Institute	Research Care And Training Program, Centre For Microbiology Research	ANDI Centre of Excellence in HIV Operational Research	**Dr Mpoke**	Eastern
*Kenya*			Pr Bukusi	
			http://www.kemri.org	
Trypanosomiasis Research Centre	Departments Of Pharmacology, Biochemistry and Primate Unit	ANDI Centre of Excellence in Pre-Clinical Development	**Dr Murilla**	Eastern
*Kenya*			http://www.kari-trc.org	
Joint Clinical Research Centre	Departments Of Clinical Services, and Research	ANDI Centre of Excellence in HIV/TB Clinical Research	**Prof Mugyenyi**	Eastern
*Uganda*			http://www.jcrc.co.ug	
Makerere University	Infectious Diseases Institute	ANDI Centre of Excellence in Epidemiology of Infectious Diseases	**Dr Coutinho**	Eastern
*Uganda*			Dr Castelnuovo	
			http://www.idi-makerere.com	
Kenya Medical Research Institute	Production Department	ANDI Centre of Excellence for Diagnostics Development and Production	**Dr Mpoke**	Eastern
*Kenya*			Dr Kimotho	
			http://www.kemri.org	
St. Luke Foundation	Kilimanjaro School of Pharmacy	ANDI Centre of Excellence in Manufacturing and Regulatory Training	**Dr Koehler**	Eastern
*Tanzania*			Dr Mlaki	
			http://www.saintlukefoundation.co.tz	
Theodor Bilharz Research Institute	Departments Of Pharmacology, Parasitology, Medicinal Chemistry, Medical Malacology, Environmental Research	ANDI Centre of Excellence on Anti-trematodal R&D	**Dr El Fandy**	Northern
			Prof Botros	
			Prof El-Sayed	
*Egypt*				
			http://www.tbri.sci.eg	
VACSERA	Units of R&D Fractionation And Venom Research, QC, Clinical Trial, R&D Microbiological, R&D Electropheresis, Toxin and Preclinical Trial	ANDI Centre of Excellence in Anti-venom Research	**Dr Tolba**	Northern
			Dr Elfiky	
*Egypt*			http://www.vacsera.com	
VACSERA	Regional Reference Lab, AFP Lab, Hepatitis Viruses Epidemiological Studies Unit, Influenza Reference Lab, Cell Culture Unit,	ANDI Centre of Excellence for virus strains diagnosis	**Dr Tolba**	Northern
			Dr Bassioni	
*Egypt*			http://www.vacsera.com	
VACSERA	Units of R&D Fractionation And Venom Research, QC, Clinical Trial, R&D Microbiological, R&D Electropheresis, Toxin and Preclinical Trial	ANDI Centre of Excellence in Anti-venom Research	**Dr Tolba**	Northern
			Dr Hamza	
*Egypt*			http://www.vacsera.com	
VACSERA	Regional Reference Lab, AFP Lab, Hepatitis Viruses Epidemiological Studies Unit, Influenza Reference Lab, Cell Culture Unit,	ANDI Centre of Excellence for virus strains diagnosis	**Dr Tolba**	Northern
			Dr Hamza	
*Egypt*			http://www.vacsera.com	
Institut Pasteur de Tunis	Departments Of Clinical Virology, Production, Medical Epidemiology, Human And Experimental Pathology, Units of Genetic Orphan Diseases Research Viral Vaccines Research And Development, Typing and Genetics Of Mycobacteria and Laboratories Of Venoms And Toxins, Vaccinology and Molecular Genetics and Immunopathology	ANDI Centre of Excellence for Bio-molecule Discovery	**Dr Louzir**	Northern
*Tunisia*			http://www.pasteur.tn	
University of Mauritius	Departments of Biomaterials and Drug Delivery/Chemistry, Biopharmaceutical /Biosciences and Molecular Biology/Biosciences	ANDI Centre for Biomedical and Biomaterials Research	**Prof Morgan**	Southern
			Dr Jhurry	
*Mauritius*			http://www.uom.ac.mu	
Council for Scientific and Industrial Research	Operating Unit Of Materials Science and Manufacturing	ANDI Centre of Excellence in Nanomedicine Research	**Dr Botha**	Southern
*South Africa*			Dr Swai	
			http://www.csir.co.za	
iThemba LABS	Departments Of Radionuclides, Radiobiology, Hadron Radiotherapy and Materials And Nanosciences	ANDI Centre of Excellence in Radiochemistry	**Dr Vilakazi**	Southern
			Dr Maaza	
*South Africa*			http://www.tlabs.ac.za	
iThemba Pharmaceuticals (Pty) Ltd	Drug Discovery	ANDI Centre of Excellence in Medicinal Chemistry	**Dr Edlin**	Southern
*South Africa*			http://www.ithembapharma.com	
South African Medical Research Council	Innovation Centre (Commercialization, Intellectual Property)	ANDI Centre of Excellence for IP management in health	**Dr Dhansay**	Southern
*South Africa*			Dr Bunn	
			http://www.mrc.ac.za	
University of Cape Town	Lung Infection and Immunity Unit	ANDI Centre of Excellence for TB Diagnostics Research	**Dr Dehda**	Southern
*South Africa*			http://www.lunginstitute.co.za/content/lung_infection.html	
University of Cape Town	IIDMM, Departments Of Chemistry, Pharmacology, Drug Discovery And Development Institute	ANDI Centre of Excellence for Drug Discovery	**Dr Price**	Southern
			Dr Chibale	
*South Africa*			http://www.uct.ac.za	
University of Cape Town	Institute Of Infectious Disease & Molecular Medicine	ANDI Centre of Excellence in Proteomics and Genomics	**Prof Hussey**	Southern
			Prof Blackburn	
			Prof Ramesar	
*South Africa*			http://www.iidmm.uct.ac.za	
University of Stellenbosch	Faculty Of Health Sciences (Centre For Infectious Diseases)	ANDI Centre of Excellence for TB Translational Research	**Dr van Zyl**	Southern
			Prof van Helden	
*South Africa*			http://www.sun.ac.za/tb	
University of Stellenbosch	Faculty Of Health Sciences (Molecule Biology & Human Genetics, Pediatrics And Child Health, Medical Microbiology, Medical Virology, Internal Medicine)	ANDI Centre of Excellence for HIV Translational Research	**Prof Nachega**	Southern
			Prof Preiser	
*South Africa*			http://www.sun.ac.za/cid	
University of the Witwatersrand	Department Of Molecular Medicine and Hematology, Antiviral Gene Therapy Research Unit	ANDI Centre of Excellence for Viral Gene Therapy	**Prof Arbuthnot**	Southern
*South Africa*			http://www.wits.ac.za/agtru/	
University of the Witwatersrand	The Wits Drug Delivery Platform	ANDI Centre of Excellence in Advanced Drug Delivery Technology	**Dr Pillay**	Southern
*South Africa*			http://www.wits.ac.za	
University of Zambia	Zambart Project	ANDI Centre of Excellence for HIV/TB Diagnostics Technologies	**Dr Ayles**	Southern
*Zambia*			Dr Muyoyeta	
			http://www.zambart.org	
African Institute of Biomedical Science & Technologies	Departments Of DMPK & Toxicology and Molecular Sciences	ANDI Centre of Excellence in in-silico Drug Metabolism & Pharmacokinetics and Toxicology Studies	**Dr Masimirembwa**	Southern
*Zimbabwe*			http://www.aibst.com	
The Biovac Institute	The Biovac Institute	ANDI Centre of Excellence in Vaccine Production	**Dr Makhoana**	Southern
			Mr. van Duyse	
*South Africa*			http://www.biovac.co.za	
Botswana Vaccine Institute	Botswana Vaccine Institute	ANDI Centre of Excellence for Vaccine Production	**Dr Matlho**	Southern
*Botswana*			http://www.bvi.co.bw	
Kwame Nkrumah University of Science and Technology	Kumasi Centre For Collaborative Research Into Tropical Medicine	ANDI Centre of Excellence for Applied Biomedical Research	**Dr van Kampen**	Western
*Ghana*			Dr Owusu-Dabo	
			http://www.kccr-ghana.org	
Noguchi Memorial Institute for Medical Research	Departments Of Clinical Pathology, Virology, Clinical Trials and Epidemiology, Parasitology, Bacteriology	ANDI Centre of Excellence in Disease Surveillance and Prevention	**Prof Nyarko**	Western
*Ghana*			http://www.noguchimedres.org	
University of Bamako	Malaria Research and Training Center, Department Of Epidemiology Of Parasitic Diseases, Faculty Of Medicine, Pharmacy And Dentistry	ANDI Centre of Excellence for Clinical Development of Malaria Products	**Prof Doumbo**	Western
			Prof Thera	
*Mali*			NA	
National Institute for Pharmaceutical R&D	Departments of Pharmaceutical Technology and Raw Material Development, Pharmacology And Toxicology, Medicinal Plant Research And Traditional Medicine, Microbiology And Biotechnology, Medicinal Chemistry/Quality Control, Human Virology/ Biotechnology and Clinic Research And Services	ANDI Centre of Excellence in Phytomedicine Research and Development	**Prof Gamaniel**	Western
*Nigeria*			http://www.niprd.org	
University of Ibadan	College Of Medicine, Malaria Research Laboratories	ANDI Centre of Excellence for Malaria Translational Research	**Prof Akinyinka**	Western
			Dr Gbotosho	
*Nigeria*			http://www.ui.edu.ng	
University of Lagos	College Of Medicine	ANDI Centre of Excellence for Malaria Diagnosis	**Prof Wole**	Western
			Dr Oyibo	
*Nigeria*			http://www.unilag.edu.ng	
LaGray Chemical Company Ltd	LaGray Chemical Company	ANDI Centre of Excellence for Drug Manufacturing	**Dr Lartey**	Western
*Ghana*			http://www.lagraychem.com	

The 38 successful ANDI Centres of Excellence in health innovation following a 4 step review process. The names of the CoE which reflects the competency of the institution as well as the contact information of head of the CoE and website are indicated.

The contact person is either the institutional head (in bold) or the lead responsible for the proposed center of excellence.

### Analysis of financing data from CoE

The applying institutions were requested to provide information on funding for their research and sources of the funding for the period 2008–2010, detailing local or external sources of funds. Local sources refer to all funds coming from within the institution or the country where the institution is located, while external funding refers to funds from outside the country or the continent, both from public and private sources.

From the 117 applications received, 93 applications (including those from the manufacturing sector) had financial data for the three-year period while data was available for either one or two years for the rest of the applications. Following the removal of duplicate funding information from institutions applying for more than one centre of excellence, a total of 88 applications were analysed. All the applications provided specific names of funding agencies on an annual basis, however, the exact amount provided by each funder or donor were not included. Therefore it is important to stress that the ranking of funding agencies or donors presented here does not represent the amount provided by each funder, but rather the frequency of appearance within all the applications received by ANDI.

All financing information were collated and analysed as follows:

i) Determination of the annual and cumulative funding over the three year period: This was done by collating the total annual research funds from applications, both with and without the applications from manufacturers.

ii) Funding sources: A listing of all the donor agencies named in each of the applications as funding the research performed was compiled with the number of times they were mentioned in the applications.

## Results and discussion

### Successful Centres of Excellence (CoE)

**Figure 2:**
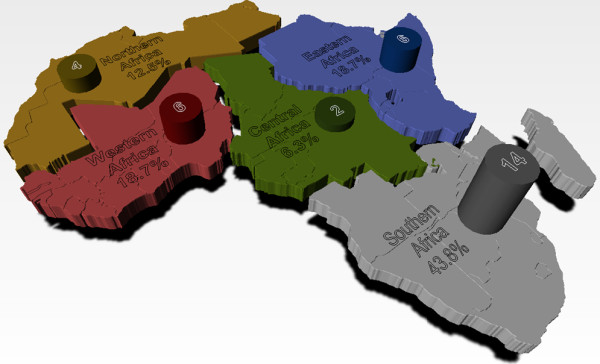
**Spread of successful Center of Excellence by region.** This figure represents the regional spread of primary 32 identified Center of Excellence, without the recognized Manufacturing CoE. The strong representation of Southern African region should be noted. Note that of the 6 manufacturers not represented in diagram, 2 came from Southern Africa and another 2 from Eastern Africa making the total number of successful centers from these regions as 16 and 8 respectively. Western Africa and Northern Africa had 1 manufacturer each making the total number of centres from these regions as 7 and 5 respectively.

The review process resulted in the identification of 32 institutions (Figure 2) plus 6 manufacturers, spread across the five African regions, that met the ANDI criteria for CoE. It should be mentioned that recognizing the manufacturers should in no way be seen as an ANDI endorsement of these manufacturers‘ capacity to produce under GMP or relevant national and international standards. Rather, the goal is to identify manufacturers that have met criteria established by ANDI, to enable them to participate in relevant ANDI network activities, including product development projects, public-private partnerships, training, etc.

The list of all the CoEs with their areas of competency and contact information are presented in Table 2. CoEs were announced during the 4^th^ ANDI stakeholders meeting in Addis Ababa, Ethiopia from 24^th^ to 27^th^ October, 2011 [26], while the finalisation of the evaluation of the manufacturing CoEs followed the meeting. A significant number of the successful CoEs are located in Southern Africa (16 in number), particularly in Republic of South Africa with a total of 12 centers, which is the highest number seen per country. The Eastern and Western African regions followed Southern Africa with 8 and 7 centers respectively, predominantly located in Kenya, Uganda, Nigeria and Ghana. Northern Africa recorded 5 CoEs of which 4 came from Egypt and one from Tunisian. It is not clear why more applications were not received from Northern Africa especially with the increasing role of Northern African region in R&D exemplified by a recent announcement from Algerian government for increased funding for research [27]. This will probably improve with better communication of future calls for applications in the Northern African region. The Central African region had the lowest number of accredited CoEs with only 2 centers located in Cameroon. The result also highlights the need for more investment and capacity building in the Central African region, particularly the Francophone countries [3,20].

It is important to mention that no one African country has the full capacity for all the components of the products value chain (Table 1), reinforcing the value of continental network with the critical mass of institutions with capacity spread across the innovation value chain [3,20,28]. With regards to academic-industry or public-private partnerships, about 38% of the CoEs claim to having active partnerships with industry. These types of public - private partnerships will further be strengthened through an integrated CoE framework programme that supports capacity building and project implementation, as further elaborated below.

### Funding of health R&D in Africa

**Figure 3:**
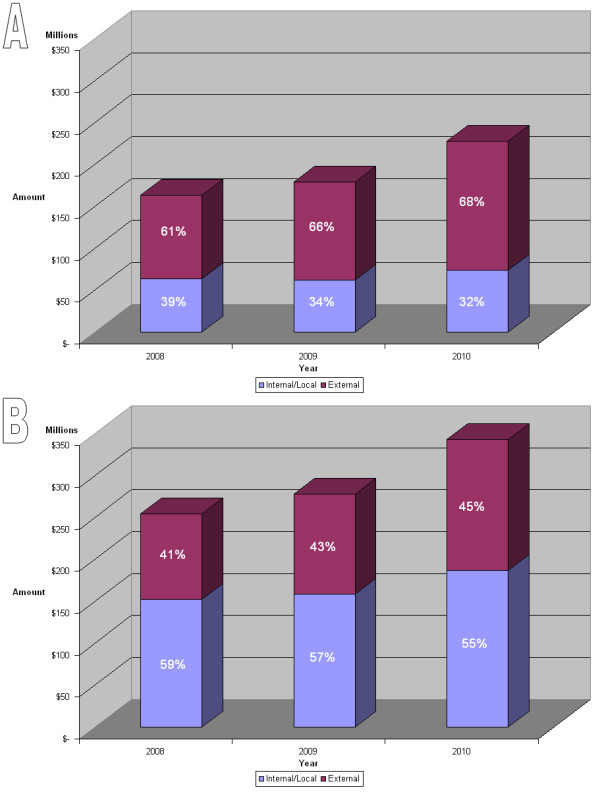
**Health R&D funding between 2008 and 2010.** Graph **A** data does not include the budget for manufacturing centers received as part of the application. On the average, 65% of R&D budget is coming from external funding sources outside the African continent. In graph **B**, the budget for manufacturers that responded to the ANDI call is added to the R&D budget on graph **A**. Note the increased internal funding to an average 57% when the budget of manufacturers is included.

The total annual research funds from all applications excluding and including manufacturers are shown in Figure 3, bar diagrams A and B respectively. The data show a steady and modest increase in annual funding over the three year period largely due to increased funding from external sources, while funding from within Africa decreased from 39% to 32% (Figure 3A). This suggests that most African countries and governments were unable to sustain investment in health R&D in the period and the hope is that this trend will now change. However, when the budget for the manufacturers are included in the analysis, the internal/external ratio was reversed with more funding coming from internal sources up to an average of 57% over the three year period (Figure 3B). This suggests that funding from both private and public sectors in Africa, largely targets manufacturing activities. Although a steady increase in funding for health innovation was observed over a three year period (Figure 3), our data highlights a number of challenges as well as opportunities for African health innovation financing. The annual budget of departments or units, within African institutions range from five hundred US Dollars ($500) to thirty five million US Dollars ($35 million) with a mean value of $1.49 million. The relatively low budget of most African institutions compared to other parts of world, could explain some of the challenges faced by African institutions in translating their research findings into usable health products. It also underlines the need for a better coordination of funding and access to grants in Africa.

**Figure 4:**
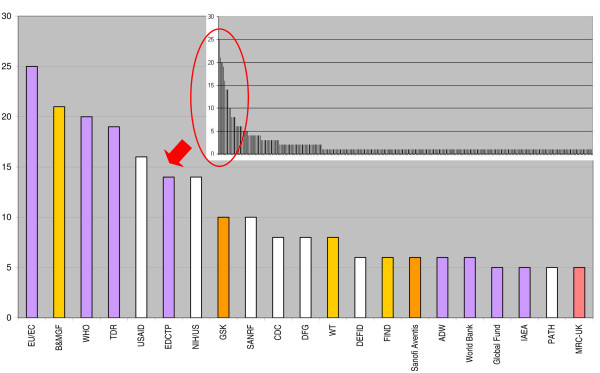
**Top 21 most frequent donors of health R&D in Africa.** The zoomed out bar graph on the right hand corner of the figure highlights the donors that were most frequently mentioned in the applications analyzed for ANDI centers of excellence. Bar colors represent the different type of funders as listed in Table 3. EU/EC (European Union and European Commission, B&MGF (Bill and Melinda Gates Foundation), WHO (World Health Organization), TDR (Special Programme for Research and Training in Tropical Diseases, USAID (United States Agency for International Development ), EDCTP (European and Developing Countries Clinical Trial Partnership), NIHUS (National Institute of Health US), GSK (GlaxoSmithKline), SANRF (South African National Research Foundation), CDC (Centre for Disease Control US), DFG (Deutsche Forschungsgeminschaft Germany), WT (Wellcome Trust), DFID (UK Department for international Development ), FIND (Foundation for Innovative New Diagnostics), ADW (Academy for Developing World), IAEA (International Atomic Energy Agency), PATH (Program for Appropriate Technologies in Health), MRC -UK (Medical Research Council UK).

A listing of all the funders/donor agencies named in each of the applications is presented in Additional file 1: Table S1. The data shows that a total of 266 different donor agencies supported research of the applying African institutions over the three year period. Out of these 266 agencies, only 27 donors were identified as African based. This financing information made it possible to further determine the 21 most frequently mentioned funding agencies in all the applications (Figure 4). The National Research Foundations (NRF) of South Africa was identified as the only African institution that made the list of 21 most frequent health research funders in Africa [29]. The fact that NRF has also been highlighted as the biggest research funding agency in Africa in the area of natural and physical sciences corroborates our data [11]. Accordingly, the Republic of South Africa also featured as one of the top 10 funders of research in Africa by country when all sources of funding are considered (Table 3). It is therefore not surprising to have identified the highest number of successful Centers of Excellence as coming from South Africa. Our data is in agreement with a trend in R&D across Africa in which institutions from countries and regions that invest more resources, as measured by the percentage of GDP invested in research and development, have a higher chance of qualifying as CoE. Countries such as Kenya, Uganda and South Africa have averaged about 1% of GDP investment in R&D [6,30]. These numbers substantiate previous publications and collaboration patterns in Africa [20].

**Table 3:** Summary of all donor information by country and other categories

**Countries**	**Public donors**						**Private and philanthropic donors**			**TOTAL FREQUENCY**
	**NA**	**A/H**	**IN**	**NPO**	**IO**	**FREQUENCY**	**IP**	**PF**	**FREQUENCY**	
United States of America	51	19	3	20		**93**	11	40	**51**	**144**
Republic of South Africa	33	2	4			**39**	4	5	**9**	**48**
United Kingdom	16	4	1			**21**	12	13	**25**	**46**
Switzerland	9	3		12		**24**	5	8	**13**	**37**
Germany	17	3	1			**21**	2	3	**5**	**26**
France	3	4	5			**12**	6		**6**	**18**
Italy	9	1				**10**	1		**1**	**11**
Sweden	9	2				**11**			**0**	**11**
Canada	6	3				**9**		1	**1**	**10**
Kenya	4	1	3			**8**		1	**1**	**9**
The Netherlands	2	2	2	1		**7**	1	1	**2**	**9**
Spain	7	1				**8**			**0**	**8**
Ghana	3	1	2			**6**			**0**	**6**
Egypt	2		2			**4**	1		**1**	**5**
Nigeria		1	1			**2**	2	1	**3**	**5**
Tanzania	1					**1**		3	**3**	**4**
Australia	1	1				**2**	1		**1**	**3**
Ireland	3					**3**			**0**	**3**
Belgium	1	1				**2**			**0**	**2**
Burkina Faso	1					**1**		1	**1**	**2**
Czech Republic	2					**2**			**0**	**2**
Finland			2			**2**			**0**	**2**
Japan						**0**	2		**2**	**2**
Norway	2					**2**			**0**	**2**
Senegal	1					**1**		1	**1**	**2**
Cuba	1					**1**			**0**	**1**
Denmark	1					**1**			**0**	**1**
Israel						**0**	1		**1**	**1**
New Zeeland						**0**		1	**1**	**1**
Republic of Saudi Arabia		1				**1**			**0**	**1**
Slovakia	1					**1**			**0**	**1**
Uganda		1				**1**			**0**	**1**
United Arabic Emirates			1			**1**			**0**	**1**
**Others**										
United Nations					66	**66**				**66**
European Union					46	**46**				**46**
Development Banks					9	**9**				**9**
African Union					4	**4**				**4**
TOTAL	186	51	27	33	125	**422**	49	79	**128**	**550**

This table show the entire data set of the different types of donors mentioned in the applications based on country and other categories such international organizational and regional organizations. NA mean National Agency, A/H = Academics & Hospitals, IN = International Network, NPO = Non-Profit Organization, IO = International Organization, IP = Industry Partner, PF = Private Foundation.

In the context of external donors, European and North American countries and agencies (including public, private and philanthropic sectors) have a strong presence in health research in Africa (Table 3). Finally, it is important to highlight the absence of emerging economies, namely China, India, South Korea and Brazil, from the list of donor countries, suggesting the need to engage these countries and to reinforce south-south partnerships with Africa.

As expressed above, the key measure of investment in R&D is the percentage of a country or region’s GDP devoted to such activities. This is often termed gross expenditure on research and development - GERD [11,13]. Available data show that Africa as a whole accounted for only 0.9% share of world GERD in 2007 while Asia had 32.2%, Oceania 1.6%, Latin America and the Caribbean 3%, Europe 27.4% and North America 38% [31]. On the other hand, some recent economic analyses have highlighted the economic growth potential of Africa - the continent's GDP rose by an average of 4.9% annually from 2000 to 2008, making it the world's third-fastest growing region [32].For this growth to be sustained, significant resources and efforts has to be invested in R&D and innovation, including through support for strong intra-African exchange and collaboration. Medical research now dominate African research, having overtaken agricultural research, which was the leading field in the 1990s [13,24]. It is important therefore to intensify and leverage this existing health R&D momentum to support collaboration among African R&D institutions to develop the most needed health products to address the continent's health needs. This will also bring economic benefits including human capital development, retention of health researchers and experts and African integration. We believe that a critical mass of CoEs in health innovation in Africa, working collaboratively and sharing information and know-how, would contribute to achieving this goal through joint projects, networking, capacity building and training as well as technology transfer and diffusion across the continent. Indeed, lessons learned from a number of international initiatives further illustrate the power of organized centers of excellence [23,33-36]. For example, Canada Network for Centres of Excellence, has helped to turn Canadian research and entrepreneurial talent into economic and social benefits for all Canadians by funding research partnerships between academia, industry, government, and not-for-profit organizations [33].

The ANDI CoEs are envisioned as a network that will bring African scientific and technical resources together to build capacity, develop and diffuse technology to address African's health challenges in a significant way [3,25,37]. A detailed project framework that defines how the ANDI COEs network will achieve the desired objectives is being finalised. For example, a structured pool of fellowships and training across the continents can be implemented through the CoE network, including MSC, PhD, postdoctoral, vocational or technical training as well as sabbatical for scientists from other African institutions, who wish to spend time at an ANDI CoE for specific training purpose. A central capacity building fund can be established to support qualifying candidates and African institutions that aspire to become a CoE in a specific field. Through this same mechanism, North–South and South-South collaboration and exchange can be enhanced. The CoE network can also support the placement of external experts who wish to spend time in an African CoE to support capacity building of Africans and gain more experience in Africa. This will include placement of experts from industry and leading academic institutions and laboratories abroad, who wish to support specific R&D, management, regulatory training and other needs of the network. Having said these, there are arguments for and against, for example the concentration of research funding in few institutions versus the distribution of academic talent and research funding among universities [38]. Indeed, the goal of ANDI is not to focus research funding on the CoEs but rather to use them to support capacity building and create partnerships to implement projects that come from a variety of African institutions.

## Conclusion

Our work has revealed the diversity and richness of African institutions and their potential to support coordinated projects and capacity building activities in areas such as disease surveillance, epidemiology, biotechnology, product development, manufacturing, as well as the development or spin off platforms for new biotechnology companies and agencies for health innovation in Africa. We believe that a credible and sustainable solution to the health challenges in Africa must leverage existing R&D, manufacturing and commercialization capacity across the continent to support integrated capacity utilization and economic development [3,20,39]. Hopefully, our work will encourage more funding for health R&D and prevent the significant fragmentation of financing in the continent. As a pan-African initiative focusing on health R&D, promoting local manufacturing and access to medicines, the ANDI initiative is in a good position to contribute in the actualization of an integrated and coordinated product R&D platform in the African continent.

## Authors’ contributions

SN conceived and generated draft paper. AO, DB, BR and FF contributed in writing the paper as well as in data collection and analysis and in figure preparation. All other authors contributed to establishing criteria and selection process as well as in reviewing draft manuscript.

## Pre-publication history

The pre-publication history for this paper can be accessed here:

http://www.biomedcentral.com/1472-698X/12/11/prepub

## Supplementary Material

Additional file 1 **Table S1.** List of all donors mentioned in the applications received. Supplementary material represents all donors names retrieved from the 117 applications received for the identification of Center of Excellence. Donors are classified (ID) by number of appearance, which means the number of times a particular donor is mentioned or acknowledged as providing funding in all applications and by respective acronym alphabetical order. Donors appearing the same number of times are having the same ranking. The table also include information regarding the type of donors and the country of origin (EU = European Union, UN = United Nations, AF = African Union). Click here for file
