# Supplementary material for: Analysis of pan-African Centres of excellence in health innovation highlights opportunities and challenges for local innovation and financing in the continent
Source: BMC Int Health Hum Rights. 2012 Jul 27;12:11. doi: 10.1186/1472-698X-12-11 (PMC3492037; doi:10.1186/1472-698X-12-11)
Supplement: Additional file 1 — Table S1. List of all donors mentioned in the applications received. Supplementary material represents all donors names retrieved from the 117 applications received for the identification of Center of Excellence. Donors are classified (ID) by number of appearance, which means the number of times a particular donor is mentioned or acknowledged as providing funding in all applications and by respective acronym alphabetical order. Donors appearing the same number of times are having the same ranking. The table also include information regarding the type of donors and the country of origin (EU = European Union, UN = United Nations, AF = African Union). [file 1472-698X-12-11-S1.doc]

Supplementary table

| **ID** | **Rank** | **Acronym** | **Type** | **Country** | **Name** | **Appearance** |
| --- | --- | --- | --- | --- | --- | --- |
| **1** | **1** | **EU/EC** | International Organization | EU | European Union and European commission | 25 |
| **2** | **2** | **B&MGF** | Private Foundation | USA | Bill & Melinda Gates Foundation | 21 |
| **3** | **3** | **WHO** | International Organization | UN | World Health Organization | 20 |
| **4** | **4** | **TDR** | International Organization | UN | Special Programme for Research and Training in Tropical Diseases | 19 |
| **5** | **5** | **USAID** | National Agency | USA | United States Agency for International Development | 16 |
| **6** | **6** | **EDCTP** | International Organization | EU | European and Developing Countries Clinical Trials Partnership | 14 |
| **7** | **6** | **NIH** | National Agency | USA | National Institutes of Health | 14 |
| **8** | **8** | **GSK** | Pharmaceutical industry | UK | GlaxoSmithKlein | 10 |
| **9** |  | **NRFSA** | National Agency | South Africa | National Research Foundation | 9 |
| **10** | **10** | **CDC** | National Agency | USA | Center of Disease Control (CDC) | 8 |
| **11** |  | **DFG** | National Agency | Germany | Deutsche Forschungsgeminschaft | 8 |
| **12** |  | **WT** | Private Foundation | UK | Wellcome Trust | 8 |
| **13** | **13** | **DFID** | National Agency | UK | Department for international Development | 6 |
| **14** |  | **FIND** | NPO | Switzerland | Foundation for Innovative New Diagnostics | 6 |
| **15** |  | **SA** | Pharmaceutical industry | France | Sanofi Aventis | 6 |
| **16** |  | **TWAS** | National Agency | Italy | The academy for developing world | 6 |
| **17** |  | **WB** | International Organization | UN | World Bank | 6 |
| **18** | **18** | **GF** | International Organization | UN | Global Fund | 5 |
| **19** |  | **IAEA** | International Organization | UN | International Atomic Energy Agency, | 5 |
| **20** |  | **MRC-UK** | National Agency | UK | UK Medical Research Council, | 5 |
| **21** |  | **PATH** | NPO | USA | PATH: a catalyst for global health | 5 |
| **22** | **22** | **DAAD** | National Agency | Germany | Deutsche Akademische Austausch Dienst | 4 |
| **23** |  | **ISP** | National Agency | Sweden | International Science Programme | 4 |
| **24** |  | **MRC-SA** | National Agency | South Africa | South African Medical Research Council, | 4 |
| **25** |  | **NEPAD** | International Organization | AF | African Union, Biosciences for Eastern and central Africa | 4 |
| **26** |  | **PFZ** | Pharmaceutical industry | USA | Pfizer | 4 |
| **27** |  | **SDC** | National Agency | Switzerland | Swiss Development Cooperation | 4 |
| **28** |  | **TIA** | National Agency | South Africa | Technology Innovation Agency | 4 |
| **29** |  | **UNEP** | International Organization | UN | United Nations Environmental Program | 4 |
| **30** |  | **VF** | Private Foundation | Switzerland | Velux Foundation, | 4 |
| **31** | **31** | **AMANET** | Private Foundation | Tanzania | The African Malaria Network Trust | 3 |
| **32** |  | **CBT** | Private Foundation | South Africa | Cape Biotech Trust | 3 |
| **33** |  | **EuroPaid** | International Organization | EU | EuroPaid, | 3 |
| **34** |  | **FF** | Private Foundation | USA | FORD FOUNDATION, | 3 |
| **35** |  | **IDRC** | National Agency | Canada | Canadian International Development and Research Centre | 3 |
| **36** |  | **IFS** | National Agency | Sweden | International Foundation of Science | 3 |
| **37** |  | **MMV** | NPO | Switzerland | Medicine for Malaria Venture | 3 |
| **38** |  | **NIAID** | National Agency | USA | National Institute of Allergy abd Infectious Diseases | 3 |
| **39** |  | **NOV** | Pharmaceutical industry | Switzerland | Novartis | 3 |
| **40** |  | **RIIP** | International network | France | International Pasteur Institutes Network | 3 |
| **41** |  | **UBSOF** | Private Foundation | Switzerland | UBS Optimus foundation, | 3 |
| **42** |  | **UNESCO** | International Organization | UN | United Nations Educational, Scientific and Cultural Organization | 3 |
| **43** |  | **USNSF** | National Agency | USA | US National Science Foundation, | 3 |
| **44** | **44** | **ADB** | International Organization | AF | African development bank, | 2 |
| **45** |  | **AECI** | National Agency | Spain | Spanish Agency for International Development and Cooperation, | 2 |
| **46** |  | **AERAS** | NPO | USA | Aeras is a non-profit product development organization dedicated to the development of effective tuberculosis | 2 |
| **47** |  | **ALC** | International network | South Africa | African Laser Centre, | 2 |
| **48** |  | **ARC** | NPO | USA | American Red Cross | 2 |
| **49** |  | **AZ** | Pharmaceutical industry | UK | Astra Zeneca | 2 |
| **50** |  | **CASR** | National Agency | Czech | Czech Academiy of Scientific Research | 2 |
| **51** |  | **CNHR** | National Agency | Kenya | Consortium for National Health Research | 2 |
| **52** |  | **DSTSA** | National Agency | South Africa | Depart of Science and Technology | 2 |
| **53** |  | **EGPAIDSF** | Private Foundation | USA | Elizabeth Glazer Pediatric AIDS Foundation, | 2 |
| **54** |  | **GTZ** | National Agency | Germany | Gesellschaft fur Technische Zusammenarbeit, | 2 |
| **55** |  | **HWU** | Academics | USA | Howard University, | 2 |
| **56** |  | **IANPHI** | International network | Finland | International Association of National Public Health Institutes | 2 |
| **57** |  | **INDEPTH** | International network | Ghana | INDEPTH-Network, | 2 |
| **58** |  | **IUATLD** | International network | France | International Union Against Tuberculosis and Lung Disease, | 2 |
| **59** |  | **LSHTM** | Academics | UK | London School of Hygiene & Tropical Medicine | 2 |
| **60** |  | **MEK** | Pharmaceutical industry | USA | Merck | 2 |
| **61** |  | **MVI** | NPO | USA | Malaria Vaccine Initiative | 2 |
| **62** |  | **NCST** | National Agency | Kenya | National Council for Science & Technology | 2 |
| **63** |  | **PEPAR** | National Agency | USA | PRESIDENT EMEGENCY PLAN FOR AIDS RELIEF USA, | 2 |
| **64** |  | **PRF** | National Agency | South Africa | Polio Research Foundation | 2 |
| **65** |  | **RPS** | National Agency | UK | Royal Pharmaceutical Society | 2 |
| **66** |  | **SAMS** | National Agency | Switzerland | Swiss Academy of Medical Sciences | 2 |
| **67** |  | **SIDA** | National Agency | Sweden | Swedish International Development Cooperation Agency | 2 |
| **68** |  | **SNSF** | National Agency | Switzerland | Swiss National Science foundation, | 2 |
| **69** |  | **STPH** | Academics | Switzerland | Swiss Tropical & Public Health Institute | 2 |
| **70** |  | **TWT** | Private Foundation | USA | Taylor Will Trust. | 2 |
| **71** |  | **UMA** | Academics | Canada | University of Manitoba, | 2 |
| **72** |  | **UNDP** | International Organization | UN | United Nation Development Program | 2 |
| **73** |  | **UNICEF** | International Organization | UN | United Nations Children's Fund | 2 |
| **74** | **74** | **AA** | Private Foundation | Burkina Faso | Association Annigoni | 1 |
| **75** |  | **AMC** | Hospital | Holland | Amsterdam Medical Centre | 1 |
| **76** |  | **AMMD** | Pharmaceutical industry | USA | American Medical Systems | 1 |
| **77** |  | **ANDRS** | National Agency | Senegal | National Agency for the Development of Research | 1 |
| **78** |  | **ANRS** | National Agency | France | Agence National pour la Recherche Scientifique | 1 |
| **79** |  | **ASRT** | National Agency | Egypt | Academy of Scientific Research and Technology | 1 |
| **80** |  | **AVAC** | NPO | USA | Global Advocacy for HIV Prevention | 1 |
| **81** |  | **AVF** | National Agency | Spain | Africa Viva Foundation | 1 |
| **82** |  | **BBSRC** | National Agency | UK | Biotechnology and Biological Sciences Research Council | 1 |
| **83** |  | **BLF** | National Agency | UK | British Lung Foundation, | 1 |
| **84** |  | **BMBV** | National Agency | Germany | Bundesministerium fur Bildung und vorschung, | 1 |
| **85** |  | **BMP** | International Organization | EU | BioMalPar | 1 |
| **86** |  | **BMS** | Pharmaceutical industry | USA | Bristol-Myers Squibb | 1 |
| **87** |  | **BNITM** | Academics | Germany | Bernard Nocht Institute for Tropical Medicine | 1 |
| **88** |  | **BP** | Pharmaceutical industry | South Africa | Batswadi Pharmaceuticals, | 1 |
| **89** |  | **CADC** | National Agency | Spain | Catalan Agency for Development Cooperation | 1 |
| **90** |  | **CAI** | Academics | AUS | Center for Advanced Imaging | 1 |
| **91** |  | **CARISA** | National Agency | South Africa | Cancer Research Initiative in South Africa | 1 |
| **92** |  | **CDG** | National Agency | Germany | Carl Duisburg-Gesellschaft | 1 |
| **93** |  | **CEDIM** | National Agency | France | French Cultural Centre | 1 |
| **94** |  | **CF** | National Agency | UK | Commonwealth Foundation, | 1 |
| **95** |  | **CGEBC** | National Agency | Cuba | Center for Genetic Eng &Biotechnology, | 1 |
| **96** |  | **CIDLID** | International network | UK | Combating Infectious Diseases of Livestock for International Development | 1 |
| **97** |  | **CIHR** | National Agency | Canada | Canadian Institute for Health Research | 1 |
| **98** |  | **CMC** | Industry | AUS | CMC Australia | 1 |
| **99** |  | **CMH** | Hospital | South Africa | Charlotte Maxeke Hospital, | 1 |
| **100** |  | **CR** | Private Foundation | UK | Comic Releif, | 1 |
| **101** |  | **CRDF** | National Agency | USA | US Civilian Research & Development Foundation | 1 |
| **102** |  | **CSR** | Academics | Switzerland | Corporate Social Responsibility | 1 |
| **103** |  | **CU** | Academics | USA | Collumbia University | 1 |
| **104** |  | **CUD** | National Agency | Belgium | Coopération Universitaire au Développement | 1 |
| **105** |  | **CWRU** | Academics | USA | Case Western Reserve University | 1 |
| **106** |  | **CWT** | National Agency | Tanzania | Concern World Tanzania, | 1 |
| **107** |  | **DA** | National Agency | Denmark | Ambassade du Danemark, | 1 |
| **108** |  | **DAHW** | National Agency | Germany | Deutsche Lepra und Tuberkulose Hilfe | 1 |
| **109** |  | **DC** | National Agency | Burkina Faso | Delegation Camillienne | 1 |
| **110** |  | **DDCF** | Private Foundation | USA | Doris Duke Charitable Foundation | 1 |
| **111** |  | **DDI** | Private Foundation | Holland | Delft Diagnostic Imaging | 1 |
| **112** |  | **DF** | National Agency | South Africa | Discovery Foundation | 1 |
| **113** |  | **DFAIT** | National Agency | Canada | Dept of Foreign Affairs and International Trade | 1 |
| **114** |  | **DNDI** | NPO | Switzerland | Drug for Neglected Diseases Initiative | 1 |
| **115** |  | **DRI** | Private Foundation | USA | DIABETES RESEARCH Institute | 1 |
| **116** |  | **DSD** | National Agency | South Africa | Diagnostic service delivery, | 1 |
| **117** |  | **EANETT** | International network | Kenya | Eastern Africa Network for Trypanosomiasis | 1 |
| **118** |  | **EDF** | International Organization | EU | European Development Fund, | 1 |
| **119** |  | **ETF** | Private Foundation | Nigeria | Educational Trust Fund | 1 |
| **120** |  | **EUANTIMAL** | International Organization | EU | EU ANTIMAL Consortium, | 1 |
| **121** |  | **FANRPAN** | International network | South Africa | Food, Agriculture and Natural Resources Policy Analysis Network | 1 |
| **122** |  | **FAO** | International Organization | UN | Food and Agriculture Organization | 1 |
| **123** |  | **FI** | Private Foundation | USA | Fetzer Institute, Kalamazoo | 1 |
| **124** |  | **FIS** | National Agency | Spain | Fondo de Investigación Sanitaria | 1 |
| **125** |  | **FRBNY** | National Agency | USA | FEDERAL RESEARCH BANK of NY | 1 |
| **126** |  | **GAIDSC** | National Agency | Ghana | Ghana AIDS Commission, | 1 |
| **127** |  | **GAVI** | NPO | Switzerland | Global Alliance, | 1 |
| **128** | **74** | **GHRDD** | National Agency | Ghana | Ghana Health Research and Development Directorate | 1 |
| **129** |  | **GU** | Academics | Sweden | Göteborg University, | 1 |
| **130** |  | **HB** | Hospital | France | Hopital Beaujon | 1 |
| **131** |  | **HmF** | Private Foundation | Germany | Humboldt Foundation | 1 |
| **132** |  | **HF** | Private Foundation | USA | Hewlett Foundation, | 1 |
| **133** |  | **HGMC** | Private Foundation | South Africa | Harmony Gold Mining Company Limited, | 1 |
| **134** |  | **HHMI** | Private Foundation | USA | Howard Hughes Medical Institute, | 1 |
| **135** |  | **HIVRT** | Private Foundation | UK | HIV Research Trust | 1 |
| **136** |  | **HRBI** | National Agency | Ireland | Health Research Board of Ireland, | 1 |
| **137** |  | **HVTN** | International network | USA | HIV Vaccine Trial Network | 1 |
| **138** |  | **IA** | National Agency | Ireland | Irish Aid, | 1 |
| **139** |  | **ICGEB** | National Agency | Italy | International Centre for Genetic Engineering and Biotechnology | 1 |
| **140** |  | **ICSUNIDO** | National Agency | Italy | International Centre for Science and High Technology | 1 |
| **141** |  | **ICTP** | Academics | Italy | The Abdus Salam ICTP | 1 |
| **142** |  | **IDB** | International Organization | BAN | Islamic Development Bank | 1 |
| **143** |  | **IDNTRA** | International network | Holland | Infectious Diseases Network for Treatment and Research in Africa, | 1 |
| **144** |  | **IDS** | Private Foundation | UK | International Donkey Santuary, | 1 |
| **145** |  | **IF** | National Agency | AUS | Innovation Fund, | 1 |
| **146** |  | **IHVN** | Academics | Nigeria | Institute for Human Virology | 1 |
| **147** |  | **ILRI** | Academics | Kenya | International Livestock Research Institute | 1 |
| **148** |  | **IMF** | Private Foundation | Kenya | Integrative Medicine Foundation, | 1 |
| **149** |  | **IMHOTEP** | International network | Egypt | Egypt-France Scientific and Technological Cooperation Program | 1 |
| **150** |  | **IMPROVED** | Pharmaceutical industry | USA | IMPROVED Pharma LLC, | 1 |
| **151** |  | **IN** | International network | EAU | INTERNATIONAL NETWORK, | 1 |
| **152** |  | **INU** | Academics | USA | INDIANA UNIVERSITY, | 1 |
| **153** |  | **IP** | Academics | France | Institut Pasteur (Lyon) | 1 |
| **154** |  | **IPM** | NPO | USA | International Partnership for Microbicide, | 1 |
| **155** |  | **ISC** | National Agency | Spain | Instituto de Salud Carlos III | 1 |
| **156** |  | **ISS** | National Agency | Italy | Instituto Superiore di Sanita | 1 |
| **157** |  | **JHU** | Academics | USA | JOHN HOPKINS UNIVERSITY | 1 |
| **158** |  | **JP** | Pharmaceutical industry | Germany | Jomaa Pharma, | 1 |
| **159** |  | **KNCV** | National Agency | Holland | KNCV Tuberculosis Foundation | 1 |
| **160** |  | **Labaid** | Private Foundation | UK | LabAid Trust | 1 |
| **161** |  | **LCB** | Private Foundation | Germany | Lutheran Church, Bavaria | 1 |
| **162** |  | **LCF** | National Agency | Spain | la Caixa Foundation | 1 |
| **163** |  | **LEU** | Academics | Belgium | Leuven University | 1 |
| **164** |  | **LGCIG** | Private Foundation | Canada | Leatherdale Global Citizen Internship | 1 |
| **165** |  | **MAD** | Pharmaceutical industry | Germany | MADAUS GmbH, | 1 |
| **166** |  | **McAF** | Private Foundation | USA | McARTHUR FOUNDATION, | 1 |
| **167** |  | **MF** | Private Foundation | USA | Microsoft Foundation, | 1 |
| **168** |  | **MFOM** | National Agency | France | Ministère Français de L’outre-Mer-France, | 1 |
| **169** |  | **MH** | Private Foundation | UK | Marlborough House | 1 |
| **170** |  | **MI** | Private Foundation | USA | Marco international, | 1 |
| **171** | **74** | **MOHP** | National Agency | Egypt | Ministry of Health and Population | 1 |
| **172** |  | **MS** | Pharmaceutical industry | Switzerland | Merck Serono | 1 |
| **173** |  | **MSF** | NPO | Switzerland | Medecin Sans Frontière | 1 |
| **174** |  | **MSH** | NPO | USA | Management Sciences for Health | 1 |
| **175** |  | **MU** | Academics | USA | Maryland university, | 1 |
| **176** |  | **NACCAP** | International network | Holland | Netherlands-African partnership for capacity development & clinical interventions against poverty-related diseases | 1 |
| **177** |  | **NATO** | International Organization | UN | North Atlantic Treaty Organization | 1 |
| **178** |  | **NC** | International Organization | EU | Nanotryp consortium | 1 |
| **179** |  | **NCMD** | Hospital | Holland | Nijmegen Center for Mitochondrial Disorders | 1 |
| **180** |  | **NCU** | Academics | UK | Newcastle University | 1 |
| **181** |  | **NFSD** | Private Foundation | Switzerland | Novartis Foundation for Sustainable Development, | 1 |
| **182** |  | **NHLS** | National Agency | South Africa | NHLS Research Trust, | 1 |
| **183** |  | **NOU** | Academics | USA | Northeastern Ohio Universities, | 1 |
| **184** |  | **NP** | Pharmaceutical industry | Nigeria | Niemeth Pharmaceuticals; | 1 |
| **185** |  | **NPRNECA** | International network | Kenya | NATURAL PRODUCT RESEARCH NETWORK FOR EASTERN AND CENTRAL AFRICA | 1 |
| **186** |  | **NRFG** | National Agency | Ghana | National Research Foundation | 1 |
| **187** |  | **NUFU** | National Agency | Norway | Norwegian Programme for Development, Research and Education | 1 |
| **188** |  | **NWU** | Academics | USA | Northwest University | 1 |
| **189** |  | **OKB** | Pharmaceutical industry | Italy | Okairos Biotech, | 1 |
| **190** |  | **OMED** | International network | Germany | World Organization of Digestive Endoscopy | 1 |
| **191** |  | **OMGE** | International network | USA | Organization Mondiale de Gastroentérologie | 1 |
| **192** |  | **OP** | Pharmaceutical industry | Nigeria | Occulus Pharmacare, | 1 |
| **193** |  | **ORI** | Pharmaceutical industry | Japan | Osato Research International, | 1 |
| **194** |  | **P&G** | Pharmaceutical industry | USA | Procter & Gamble | 1 |
| **195** |  | **PADIP** | NPO | USA | PneumoADIP | 1 |
| **196** |  | **PF** | Private Foundation | USA | PACKARD FOUNDATION, | 1 |
| **197** |  | **PHA** | Pharmaceutical industry | South Africa | Pharmaco | 1 |
| **198** |  | **PHAC** | National Agency | Canada | Public Health Agency Canada | 1 |
| **199** |  | **PHEA-ETI** | International network | Kenya | Partnership for Higher Education in Africa (PHEA) Educational Technology Initiative (ETI) | 1 |
| **200** |  | **PHF** | National Agency | USA | Public Health Foundation, | 1 |
| **201** |  | **PI** | NPO | USA | Pathfinder International, | 1 |
| **202** |  | **PMI** | NPO | USA | President Malaria Initiative | 1 |
| **203** |  | **PRU** | Academics | South Africa | Pretoria University | 1 |
| **204** |  | **PSF** | National Agency | South Africa | Patent Support Fund, | 1 |
| **205** |  | **PSI** | National Agency | Switzerland | Paul Scherrer Institute | 1 |
| **206** |  | **PUU** | Academics | USA | Purdue University, | 1 |
| **207** |  | **RCN** | National Agency | Norway | RC of Norway, | 1 |
| **208** |  | **RDIS** | National Agency | Ireland | Irish Research Council for the Humanities and Social Sciences | 1 |
| **209** |  | **RFF** | Private Foundation | USA | Rocker Feller Foundation | 1 |
| **210** |  | **RIK** | Pharmaceutical industry | Japan | RIKEN Institute | 1 |
| **211** |  | **RMFF** | Private Foundation | USA | Ralph and Marion Falk Foundation, | 1 |
| **212** | **74** | **RP** | Pharmaceutical industry | Switzerland | Roche Pharmaceuticals | 1 |
| **213** |  | **RPDP** | National Agency | South Africa | Researcher and Professionals Development Programme | 1 |
| **214** |  | **RTI** | National Agency | Holland | Royal Tropical institute | 1 |
| **215** |  | **SAAIDSVI** | National Agency | South Africa | South African AIDS Vaccine Initiative, | 1 |
| **216** |  | **SACA** | National Agency | South Africa | South African Cancer Association, | 1 |
| **217** |  | **SAIF** | National Agency | South Africa | South African Innovation Fund | 1 |
| **218** |  | **SAMI** | National Agency | South Africa | South African Malaria Initiative | 1 |
| **219** |  | **SANBio** | International network | South Africa | Southern African Network for Biosciences, | 1 |
| **220** |  | **SAPRF** | National Agency | South Africa | South African Poliomyelitis Research Foundation, | 1 |
| **221** |  | **SAU** | Academics | Sweden | Sahlgrenska University | 1 |
| **222** |  | **SCUS** | Private Foundation | USA | Save the Children US, | 1 |
| **223** |  | **SEYA** | Private Foundation | NZ | SEYA Investments Ltd, | 1 |
| **224** |  | **SFH** | International network | Nigeria | SOCIETY FOR FAMILY HEALTH | 1 |
| **225** |  | **SG** | National Agency | Slovakia | Slovak Government | 1 |
| **226** |  | **SHB** | Pharmaceutical industry | South Africa | Shimoda Biotech, | 1 |
| **227** |  | **SSI** | Industry | South Africa | SSI-DHV | 1 |
| **228** |  | **SSP** | Academics | Uganda | Sewankambo Scholarship Project | 1 |
| **229** |  | **ST** | Pharmaceutical industry | USA | Sigma-Tau | 1 |
| **230** |  | **SWB** | NPO | USA | Scientists without Borders, | 1 |
| **231** |  | **TA** | Private Foundation | Senegal | Trust Africa | 1 |
| **232** |  | **TBT** | Pharmaceutical industry | Holland | Tibotec | 1 |
| **233** |  | **THI** | NPO | USA | The Hib Initiative, | 1 |
| **234** |  | **THRIP** | National Agency | South Africa | Technology and Human Resources for Industry Programme | 1 |
| **235** |  | **TPAF** | NPO | Holland | The Pharm Access Foundation, | 1 |
| **236** |  | **UAEA** | Academics | RSA | Abd-El Aziz University -Saudi Arabia/ American University, | 1 |
| **237** |  | **UBA** | Academics | Spain | University of Barcelona | 1 |
| **238** |  | **UBO** | Academics | Germany | University of Bonn, | 1 |
| **239** |  | **UC** | Academics | USA | University of Cincinnati | 1 |
| **240** |  | **UCH** | Academics | USA | UNIVERSITY OF CHICAGO, | 1 |
| **241** |  | **UCL** | Academics | UK | University College London (UCL), | 1 |
| **242** |  | **UCSD** | Academics | USA | University of California San Diego, | 1 |
| **243** |  | **UCSF** | Academics | USA | University of California San Francisco | 1 |
| **244** |  | **UFH** | Academics | Ghana | University of Fort Hare, | 1 |
| **245** |  | **UFO** | Academics | USA | UNIVERSITY OF FORTHARE | 1 |
| **246** |  | **ULY** | Academics | France | Lyon university -France | 1 |
| **247** |  | **UMI** | Academics | USA | University of Michigan | 1 |
| **248** |  | **UMI** | Academics | USA | University of Minnesota | 1 |
| **249** |  | **UMM** | Academics | France | Université de la Méditerranée | 1 |
| **250** |  | **UNFPA** | International Organization | UN | United Nations Population Fund | 1 |
| **251** |  | **UNITAID** | International Organization | UN | UNITAID | 1 |
| **252** |  | **UPI** | Academics | USA | University of Pittsburg, | 1 |
| **253** |  | **USCRDF** | National Agency | USA | USCRDF Civilian Research and Development Foundation, | 1 |
| **254** |  | **USEJR** | International network | Egypt | US-Egypt Joint Research, | 1 |
| **255** |  | **UWA** | Academics | USA | University of Washington | 1 |
| **256** |  | **UWI** | Academics | Canada | University of Winnipeg | 1 |
| **257** |  | **VAC** | Pharmaceutical industry | Egypt | Vacsera | 1 |
| **258** | **74** | **VALHP** | Private Foundation | South Africa | Value Added Life Health Products, | 1 |
| **259** |  | **VSHD** | NPO | USA | Venture Strategies Health Development | 1 |
| **260** |  | **VT** | Industry | Israel | Protea Vaccine Technologies | 1 |
| **261** |  | **VWF** | Private Foundation | Germany | Volkswagen Foundation | 1 |
| **262** |  | **WF** | International Organization | UN | World Federation | 1 |
| **263** |  | **WGC** | International Organization | UN | World Gold Council, | 1 |
| **264** |  | **WHU** | Academics | Germany | Witten University /Herdeke | 1 |
| **265** |  | **WLF** | International network | USA | World Lung Foundation | 1 |
| **266** |  | **WRAIR** | National Agency | USA | Walter Reed Army Institute of Research | 1 |
